# Tumor Suppression by RNA from C/EBPβ 3′UTR through the Inhibition of Protein Kinase Cε Activity

**DOI:** 10.1371/journal.pone.0016543

**Published:** 2011-01-24

**Authors:** Ying Wang, Da-Quan Sun, Ding-Gan Liu

**Affiliations:** State Key Laboratory of Molecular Biology, Institute of Biochemistry and Cell Biology, Shanghai Institutes for Biological Sciences, Chinese Academy of Sciences, Shanghai, China; University Health Network, Canada

## Abstract

**Background:**

Since the end of last century, RNAs from the 3′untranslated region (3′UTR) of several eukaryotic mRNAs have been found to exert tumor suppression activity when introduced into malignant cells independent of their whole mRNAs. In this study, we sought to determine the molecular mechanism of the tumor suppression activity of a short RNA from 3′UTR of C/EBPβ mRΝΑ (C/EBPβ 3′UTR RNA) in human hepatocarcinoma cells SMMC-7721.

**Methodology/Principal Findings:**

By using Western blotting, immunocytochemistry, molecular beacon, confocal microscopy, protein kinase inhibitors and *in vitro* kinase assays, we found that, in the C/EBPβ 3′UTR-transfectant cells of SMMC-7721, the overexpressed C/EBPβ 3′UTR RNA induced reorganization of keratin 18 by binding to this keratin; that the C/EBPβ 3′UTR RNA also reduced phosphorylation and expression of keratin 18; and that the enzyme responsible for phosphorylating keratin 18 is protein kinase Cε. We then found that the C/EBPβ 3′UTR RNA directly inhibited the phosphorylating activity of protein kinase Cε; and that C/EBPβ 3′UTR RNA specifically bound with the protein kinase Cε-keratin 18 conjugate.

**Conclusion/Significance:**

Together, these facts suggest that the tumor suppression in SMMC-7721 by C/EBPβ 3′UTR RNA is due to the inhibition of protein kinase Cε activity through direct physical interaction between C/EBPβ 3′UTR RNA and protein kinase Cε. These facts indicate that the 3′UTR of some eukaryotic mRNAs may function as regulators for genes other than their own.

## Introduction

A malignant tumor is caused by a series of abnormal expressions and/or deviant functions of genes governing cell proliferation and differentiation (including proto-oncogenes and tumor suppressor genes). The protein kinase Cε (PKCε) is an oncogene important in tumorigenesis [1], [2]. PKCε has been classified as a novel PKC isotype and is characterized as calcium-independent and phorbol ester/diacylglycerol-sensitive. A characteristic of PKCε is that it binds a large number of interacting proteins, indicating the generality of its actions. It is activated in the cytoplasm by diacylglycerol or phorbol esters, and it phosphorylates downstream target molecules, thereby transducing growth signals into the nucleus to promote gene expression [3]. PKCε specifically binds and phosphorylates keratin 18 (CK18), a component of the cellular intermediate filaments [4].

Abnormal, tumoral growth of cells is suppressed by the genes regulating oncogenes or oncogene-related genes, *i.e.*, tumor suppressor genes. Tumor suppressor genes can be classified by their various functions, such as those which operate as transcription factors, DNA damage sensors, cell cycle checkpoint controllers, etc. [5]. In recent years many micro-RNAs have been found to function as tumor suppressors. Generally, a tumor suppression effect may be exerted by any cellular factor inhibiting the genes responsible for abnormal cell growth. Therefore, tumor suppressors may theoretically be any of a variety of cellular factor, or even isolated functional parts of gene expression products.

The 3′UTR of eukaryotic mRNA, referred to *in this study* as the RNA segment between the final stop codon and the poly A tail, is a well-known regulation region for its own mRNAs. 3′UTR regulates, possibly by interacting with miRNA, the mRNA stability, nuclear export, translation efficiency, subcellular localization, and time of translation [6]–[8]. Since the last century, several RNAs from 3′UTRs (referred hereafter to as 3′UTR or 3′UTR RNA) have been found to exert tumor suppression activity when introduced into malignant cells as isolated segments. These include α-tropomyosin 3′UTR [9], ribonucleotide reductase subunits R1 and R2 3′UTRs [10], putative polycomb gene mel-18 3′UTR [11], prohibitin 3′UTR [12] and the C/EBPβ 3′UTR treated in this study. It is notable that these 3′UTRs suppress tumors independently from their mRNAs. For α-tropomyosin 3′UTR, the growth inhibition was explained as a result of the activation of a double strand RNA-dependent protein kinase (PKR), leading to the inhibition of overall protein synthesis [13]. Significantly, the 3′UTR of PTENP1, a pseudogene homologous to the tumor suppressor gene PTEN, was found to exert tumor suppressor activity though removing some miRNA that down-regulates the expression of PTEN, thus liberating the expression of the latter [14]. However, the molecular mechanisms behind the functions of the other tumor suppressive 3′UTRs so far remain unclear. That the 3′UTRs may act as regulators for genes other than their own (trans-regulators) is a possibility which cannot be ruled out [15].

**Figure 1:**
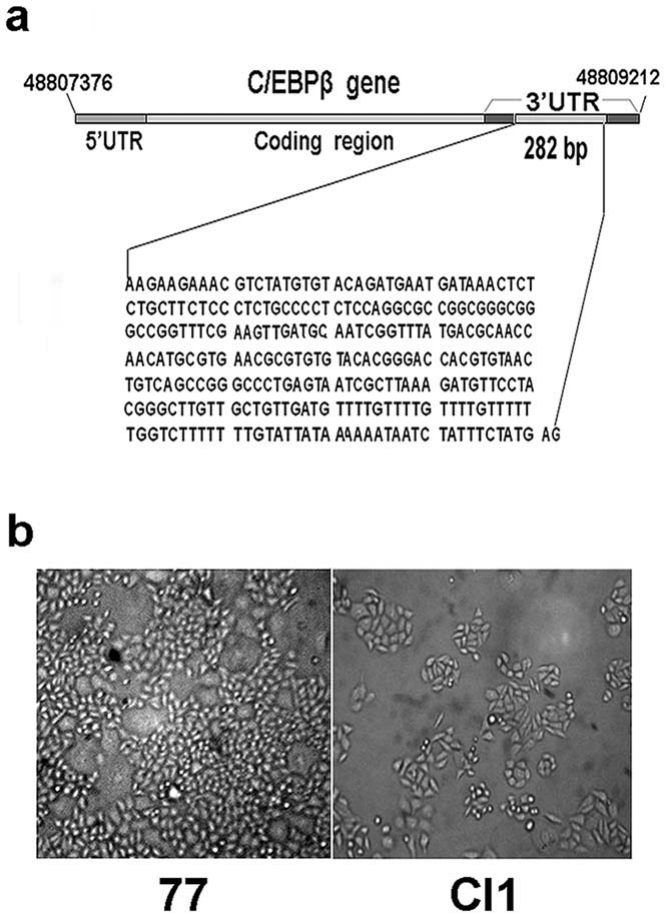
C/EBPβ 3′UTR, SMMC-7721 and Cl1 cells. (**a**) Position of the C/EBPβ gene on the human genome and the location of C/EBPβ 3′UTR (0.28 kb). (**b**) Morphology of SMMC-7721 (77) and Cl1 cells. Bars, 50 µm.

From 1991–1992, in an attempt to search for any gene with the potential for tumor suppression by transfection of malignant DT cells [16] with a pcD2 plasmid library of normal human cDNAs [17], we [18] found a pcD2 plasmid containing a 0.5kb cDNA insert (called p14-6), which, upon stable transfection, induced phenotypic reversion in a portion of the DT cells. The 0.5kb cDNA insert was sequenced [19] and was found to be the middle section of the 3′UTR of the transcription factor C/EBPβ (also named NF-IL6) mRNA [20]. When linker sequences were removed, the cDNA or RNA segment was 282 bases long (Fig. 1). This RNA segment will be referred to thereafter as C/EBPβ 3′UTR or C/EBPβ 3′UTR RNA.

In recent years, our group has continued to study the molecular mechanism of the tumor suppression function of C/EBPβ 3′UTR. We tested the C/EBPβ 3′UTR to see if it is tumor suppressive in other cultured tumor cell lines. We found that the stable transfection of p14-6 plasmid into SMMC-7721 hepatoma cells led to a significant decrease in the malignancy of a large portion of transfectants [21]. SMMC-7721(7721) is a highly malignant hepatocarcinoma cell line established from a surgically excised specimen of a Chinese hepatocellular carcinoma patient [22], [23]. About 70% of C/EBPβ 3′UTR-stably transfected 7721 cell colonies were revertants, *i.e*., transfectants with reduced malignancy, as measured by soft agar and nude mice tumorigenicity tests [21]. A selection of these revertant colonies was cloned and characterized. Their cell morphology was found to be flatter than that of 7721, especially at low density in culture (Fig. 1). Hybridizations have shown that, in these revertants, the transfected C/EBPβ 3′UTR is highly expressed, while the expression of endogenous full-length C/EBPβ mRNA has not been markedly changed [21].

We then selected a typical revertant cell strain, Cl1 [21], [24], from the C/EBPβ 3′UTR-stably transfected 7721 cells, for detailed molecular biological study. A cDNA microarray analysis of the Cl1 gene expression profile [21] revealed that tens of genes favorable for phenotypic reversion had been up-regulated, and several genes related to malignancy, including CK18, been down-regulated. Given that no protein is translated from C/EBPβ 3′UTR, its tumor suppression effect could have been achieved only by its interactions with cellular factors. In fact, we have found that C/EBPβ 3′UTR specifically bound CK18 [25].

We therefore investigated the effects of C/EBPβ 3′UTR in the reorganization of CK18. At the same time, we investigated the phosphorylation state of CK18 and the effect of C/EBPβ 3′UTR on this phosphorylation. In this work, we show that CK18 filaments are reorganized in the Cl1 cells; a portion of the Cl1 cell population is delayed at the S and G2/M phases of their cell cycle; and the average phosphorylated CK18 (pCK18) and the total amount of CK18 are lower in Cl1 than in 7721. Furthermore, we show that the enzyme responsible for CK18 phosphorylation is PKCε, and that the lower phosphorylation resulted from the inhibition of PKCε activity. Subsequently, we have identified the direct inhibition of PKCε activity by C/EBPβ 3′UTR RNA, and have found that C/EBPβ 3′UTR RNA specifically formed a complex with the CK18-PKCε conjugate. Therefore, the suppression of 7721 hepatocarcinoma cell growth is caused by the inhibition of PKCε by C/EBPβ 3′UTR RNA *via* its interaction with PKCε and CK18.

## Results

### C/EBPβ 3′UTR transfection does not affect the expression of C/EBPβ protein

As all the experiments presented in this article were performed in Cl1 cells, these shall be described again in detail. Cl1 [21] is a revertant cell strain derived from the 7721 human hepatocarcinoma cell line by the stable transfection of C/EBPβ 3′UTR cDNA plasmid, p14-6 [18]. The nucleotide sequence of C/EBPβ 3′UTR cDNA, as well as the cell morphology of Cl1 and its malignant original, 7721, are shown in Fig. 1. The transfected, exogenous C/EBPβ 3′UTR cDNA is overexpressed in Cl1 [21], and its colony-forming ability in soft agar and tumorigenicity in nude mice, both important measures for malignancy, were significantly reduced compared to 7721 [21].

**Figure 2:**
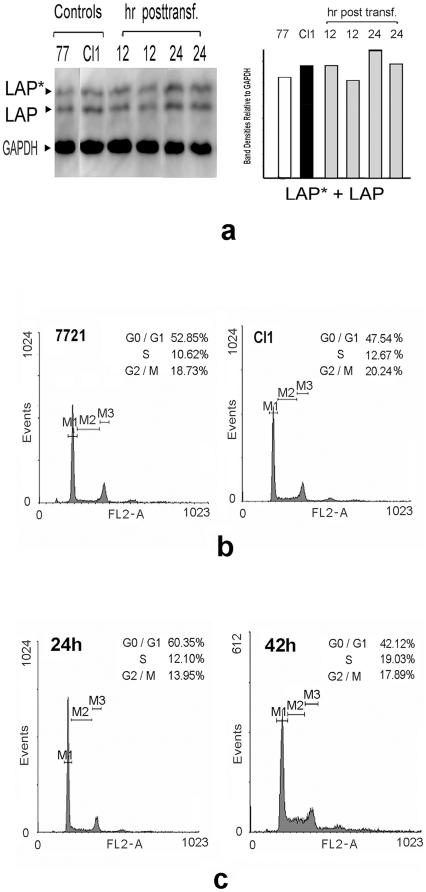
C/EBPβ proteins expression, and flow cytometric analysis of SMMC-7721, Cl1 cells and C/EBPβ 3′UTR-transfectnants. (**a**) Western blot against C/EBPβ proteins (LAP* and LAP were detected) in SMMC-7721, Cl1 and C/EBPβ 3′UTR RNA-transfected SMMC-7721 cells. The gel photograph is a representative experiment, and the histogram shows the average values of the total C/EBPβ protein amounts, measured by band density scanning. (**b**) Flow cytometry histogram of SMMC-7721 and Cl1 cells. (**c**) Flow cytometry histogram of SMMC-7721 cells transfected with C/EBPβ 3′UTR RNA, at 24 h and 42 h post-transfection.

A possible mechanism for the suppression of tumors by 3′UTR is its effect on tumoral gene expression *via* titrating out the regulating miRNA [14]. Our previous cDNA array results for Cl1 [21], however, did not show any definite change in the C/EBPβ mRΝΑ level in these transfectant cells, in which the exogenous C/EBPβ 3′UTR was overexpressed. Results of preliminary Western blotting of C/EBPβ proteins were in agreement with the above (unpublished). To verify whether or not exogenous C/EBPβ 3′UTR influenced endogenous C/EBPβ protein synthesis in Cl1 cells, we performed Western blots of Cl1 and C/EBPβ 3′UTR RNA-transiently transfected 7721 cells, using an antibody against C/EBPβ proteins. Results showed that the main C/EBPβ protein isoforms in 7721 and Cl1 cells were LAP*(the largest C/EBPβ protein isoform, at about 38 kDa) and LAP (at about 35 kDa), and that total amounts of C/EBPβ isoform proteins did not vary noticeably on average (Fig. 2a). Therefore, C/EBPβ 3′UTR did not affect the expression of the full-length C/EBPβ mRNA, indicating a difference in mechanism with the PTENP1 tumor suppressor gene pseudogene.

### C/EBPβ 3′UTR transfection causes delay of cell cycle at the S and G2/M phases

Cl1 grows much more slowly than 7721, and forms only very small tumors in nude mice [21], [24]. Thus, the cell cycle of Cl1 has also slowed down, as was confirmed though flow cytometric analysis (Fig. 2b). We found increased cell populations for Cl1 at the S phase, and a slight increase in cell population at the G2/M phase. The transient transfection by C/EBPβ 3′UTR RNA also resulted in a similar retardation of the cell cycle progression, and the retardation was positively related to time posttransfection up to 42 h, thus indicating that the C/EBPβ 3′UTR was responsible for this delay (Fig. 2c).

### C/EBPβ 3′UTR causes CK18 filament reorganization by binding to CK18

**Figure 3:**
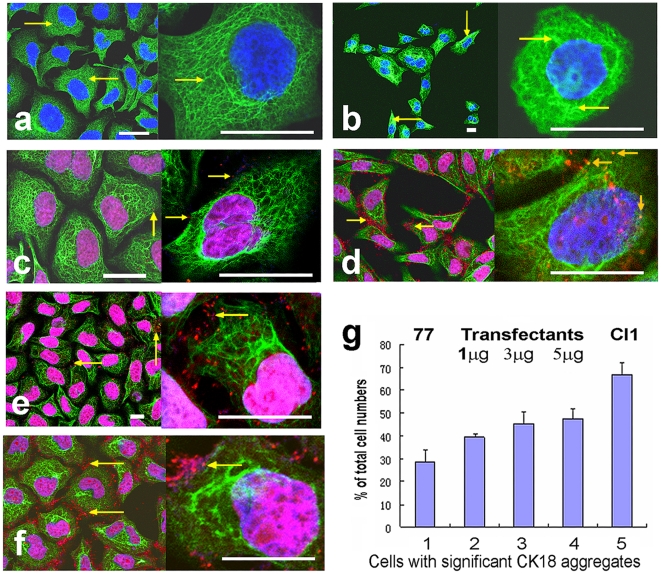
C/EBPβ 3′UTR RNA binds CK18 and alters its intracellular organization in SMMC-7721 and Cl1 cells. Confocal micrographs of the cells immunostained with a fluorescein-labeled antibody against CK18 (green), and of the cells immunostained as above and probed with a fluorescent-labeled molecular beacon specific to C/EBPβ 3′UTR RNA (red dots). (**a**) SMMC-7721 cells. Arrows indicate the thin CK18 network in the cytoplasm. (**b**) Cl1 cells. Arrows indicate the aggregates and bundles of CK18. (**c**) SMMC-7721 cells probed with the molecular beacon for C/EBPβ 3′UTR RNA. Arrows indicate the molecular beacon binding with C/EBPβ 3′UTR RNA on the CK18 filaments. (**d**) Cl1 cells probed with the same molecular beacon. Note the greater amount of red fluorescence from the molecular beacon than in SMMC-7721, and the positioning of the fluorescence both around the CK18 filaments and even on the CK18 aggregates (the orange arrows in the larger amplification). (**e**) SMMC-7721 cells transfected with 1 µg/well of C/EBPβ 3′UTR RNA and probed with the same molecular beacon. Arrows indicate the fluorescence of the molecular beacon on the CK18. (**f**) SMMC-7721 cells transfected with 5 µg/well of C/EBPβ 3′UTR RNA and probed with the same molecular beacon. Arrows indicate the fluorescence of the molecular beacon on the CK18. Bars, 10 µm. (**g**) Percentage amounts of cells with significant CK18 aggregates in total cell populations of SMMC-7721, Cl1 and SMMC-7721 transfected with varying amounts of C/EBP 3′UTR RNA. Cells were grown in 24-well plates. 1, SMMC-7721. 2-4, SMMC-7721 transfected with 1, 3, 5 µg/well of C/EBP 3′UTR RNA respectively. 5, Cl1.

Given that keratins are important components of the cytoskeleton, the flat cell morphology may well be attributed to alterations in keratin organization. We performed laser confocal microscope observations on immunocytochemically fluorescent-stained cell lines. The 7721 cells showed thin and dense CK18 networks (green fluorescence) over almost all of their cytoplasm (Fig. 3a, arrows). In the Cl1 cells, however, as well as the thin CK18 filaments, there were many CK18 filaments forming aggregates or bundles. Although such aggregates do exist in 7721 cells, their number in Cl1 is much greater than in the former (Fig. 3b, arrows).

Given that Cl1 cells differ from 7721 cells only in the presence of transfected C/EBPβ 3′UTR, and that C/EBPβ 3′UTR did specifically bind CK18 *in vitro* [25], the 3′UTR might be involved in the formation of CK18 aggregates. To prove this, we used a red fluorescent molecular beacon, which is specific to C/EBPβ 3′UTR, to detect whether it was co-localized with the CK18 aggregates. The micrographs confirmed this: in 7721 cells probed with the molecular beacon, there is only a little dot-like red fluorescence, showing that there is no exogenous C/EBPβ 3′UTR RNA within the cells (Fig. 3c). In Cl1 cells with highly expressed C/EBPβ 3′UTR RNA, however, a large amount of dot-like molecular beacon fluorescence was found located mainly in the vicinity of CK18 networks and aggregates, and even on the aggregates (Fig. 3d), indicating that C/EBPβ 3′UTR RNA had bound with the CK18. Control 7721 cells, which had been transfected with C/EBPβ 3′UTR RNA, also showed a large amount of molecular beacon fluorescence in the vicinity of CK18 (Fig. 3e,f). To make an estimation of the number of cells with significant CK18 aggregates and to further confirm the relationship between CK18 aggregates and C/EBPβ 3′UTR RNA, we transfected 7721 cells with different amounts of C/EBPβ 3′UTR RNA, and then calculated [26] the percentage values of the numbers of cells with significant CK18 aggregates in the total cell populations. The results showed that the percentage ratios of the numbers of cells with significant CK18 aggregates increased with the increase of transfecting C/EBPβ 3′UTR RNA doses (Fig. 3g). Therefore, the altered CK18 organization in Cl1 was attributed to the overexpressed C/EBPβ 3′UTR RNA binding itself to the CK18. This is in accordance with our previous findings [25].

### Reduction of amounts of pCK18 and total CK18 in Cl1 cells

**Figure 4:**
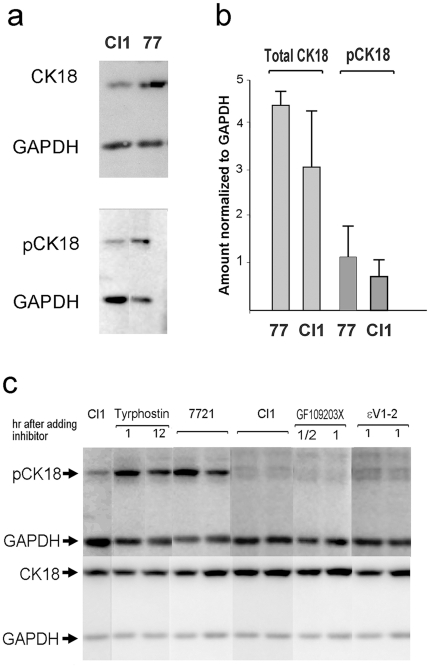
Amounts of pCK18 and total CK18 in Cl1 cells decreased; the responsible enzyme is PKCε. (**a**) Typical examples of Western blots for pCK18 and CK18. (**b**) Histogram of data of band densities comparing the amounts of pCK18 and CK18 in the two cell lines, calculated from more than five independent Western blot experiments. (**c**) Protein kinase inhibition experiments. Individual components of a kinase inhibitor library were used to treat cultured cells for definite times. Then the cells were lysed and subjected to Western blotting for pCK18 and CK18. Typical Western blots, including a protein tyrosine kinase inhibitor (tyrphostin), a general PKC inhibitor (GF109203X), and a PKCε-specific inhibitor (εV1-2), are combined. Untreated Cl1 and SMMC-7721 cells were used as controls.

Using antibodies against total CK18 or pCK18, we checked the phosphorylated states of CK18 in the 7721 and Cl1 cells with Western blotting. We found that there appeared to be less pCK18 in Cl1 cells than in 7721 cells. To verify this, a series of repeated Western blots was performed, and the band densities were quantitatively measured using Multi-Gauge version 3.0 software (FujiFilm). Results from more than five experiments were calculated and normalized to the density of glyceraldehyde 3-phosphate dehydrogenase (GAPDH) bands, which served as an inner control. The results showed that the average amounts of pCK18 and of total CK18 in the Cl1 cells were less than in 7721 (Fig. 4a,b). The reduction in amount (expression) of total CK18 in Cl1 is in accordance with the cDNA array results stating that keratin 18 is down-regulated [21]. It is well known that the phosphorylation status, as well as the total amount of a protein often change during different stages of cell cycle, cell growth and proliferation. In this case, however, the decrease of phosphorylated CK18 and total CK18 do not seem to be dependent on the cell cycle or cell proliferation, as in all repeated experiments the cells were cultured under routine conditions without adding any factor affecting cell cycle or proliferation etc. Thus the reduction of pCK18 in Cl1 suggests that some protein kinase responsible for the phosphorylation of CK18 might be inhibited by the C/EBPβ 3′UTR RNA. Therefore, we then identified the protein kinase responsible for CK18 phosphorylation, and the effects of C/EBPβ 3′UTR RNA on that kinase.

### The protein kinase responsible for CK18 phosphorylation is PKCε

A PKCε-like kinase has been reported to bind with and phosphorylate CK18 [4]. However, in this case, confirmation was still required to determine whether the kinase responsible for CK18 phosphorylation was really PKCε. For this purpose, we utilized a library of small-molecular kinase inhibitors. We performed a series of cell cultures in multiwell plates, in which respective kinase inhibitors were added to the culture media. After scheduled times, the cells were collected, lysed, separated by PAGE, and subjected to Western blots against total CK18 and pCK18. The results (a combined figure of representative experiments is shown in Fig. 4c) showed that only PKC-specific kinase inhibitors were effective in reducing the density of bands of pCK18. When a PKCε-specific inhibitor, εV1-2 [27], was used, significant reduction in band density of pCK18, almost equal to the reduction induced by general PKC inhibitors, was observed. Therefore, the enzyme responsible for CK18 phosphorylation was found to be PKCε, indicating that C/EBPβ 3′UTR RNA was capable of interacting with PKCε to lower CK18 phosphorylation.

### C/EBPβ 3′UTR directly inhibits PKCε in *in vitro* enzymatic activity assay

**Figure 5:**
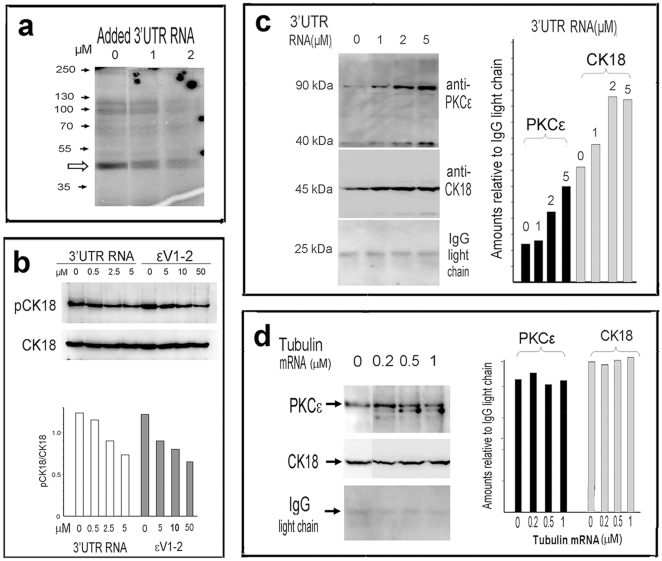
C/EBPβ 3′UTR directly inhibits the activity of PKCε and specifically binds CK18-PKCε conjugate. (**a**) Preliminary test of the activity of calcium-independent PKCs. The hollow arrow indicates a 45 kDa labeled band (probably CK18). (**b**) C/EBPβ 3′UTR RNA directly inhibits PKCε activity. A representative Western blot is shown, with the band densities expressed as the ratios pCK18/CK18 quantitatively displayed in the histogram. (**c**) RNA binding-coupled co-immunoprecipitation showing that C/EBPβ 3′UTR RNA forms a complex with CK18 and PKCε. The quantitative histogram indicating the amounts of CK18 and PKCε bands is included. (**d**) Control RNA binding-coupled co-precipitation, in which β-tubulin RNA (1.4 kb) was used instead of C/EBPβ 3′UTR RNA. The quantitative histogram showing the amounts of PKCε and CK18 bands is shown beside it. In this experiment the antibody against PKCε detected a smaller molecule that seems to bind tubulin RNA; but its identity is unknown.

As PKCε was the protein kinase that phosphorylated CK18, and given that the amount of pCK18 in the Cl1 cells had been reduced, it was necessary to determine whether the activity of PKCε could be inhibited by C/EBPβ 3′UTR RNA. Preliminarily, we checked the activity of the calcium-independent PKC isotypes in 7721 cells with γ-^32^P-labeled ATP and EGTA. Results showed that ^32^P-labeled cellular proteins were gradually reduced with an increase in the amounts of C/EBPβ 3′UTR RNA added to the reaction mixture (Fig. 5a).

We then reconfirmed our finding that the phosphorylation of CK18 was reduced by exogenous C/EBPβ 3′UTR RNA. The assay was done using total cell lysate. In order to exclude the contributions of other protein kinases existing in the cell lysate and to determine whether the kinase activity was due to protein kinase Cε, the PKCε-specific peptide inhibitor εV1-2 was used as a control, and EGTA was present in the kinase assay mixture to inhibit the activity of calcium-dependent protein kinases. Under these experimental conditions, the detected kinase activity that was sensitive to εV1-2 inhibition could be ascribed largely to the protein kinase Cε.

The source of PKCε, a 7721 cell extract, was prepared. To increase PKCε activity, phorbol myristate acetate (PMA) was added to the extract. The cellular CK18 was enriched from another 7721 cell extract, prepared using Bronfman's method [28]. The PKCε source extract was mixed with the CK18-enriched extract and EGTA, as described in Materials and Methods, and increasing amounts of C/EBPβ 3′UTR RNA (for the experimental group) or εV1-2 (for the control group) were added in the reaction mixtures, respectively. After a 20–25 min incubation at 30°C, the samples were separated on PAGE, and subsequent Western blots were performed for CK18 and pCK18. Repeated experiments showed that the amounts of pCK18 tended to negatively correlate with the amounts of C/EBPβ 3′UTR RNA added to the reaction mixture. The control εV1-2 showed a very similar tendency. A representative result is shown in Fig. 5b. These results showed unambiguously that C/EBPβ 3′UTR RNA directly inhibited PKCε activity.

### C/EBPβ 3′UTR forms a complex with PKCε-CK18 conjugate in RNA binding-coupled immunoprecipitation

Aside from binding and phosphorylating CK18, PKCε also forms signaling modules through physical interactions with signal transducing molecules [29]. As C/EBPβ 3′UTR RNA specifically binds CK18 *in vitro* and *in vivo*, it is possible for these three molecules (C/EBPβ 3′UTR RNA, CK18 and PKCε) to interact with one another to form a complex. To check this possibility, we performed an RNA binding-coupled immunoprecipitation experiment. 7721 cell extracts were divided into equal aliquots, and equal volumes of C/EBPβ 3′UTR RNA or tubulin RNA (a 1.4 kb fragment of coding region) both in increasing concentrations, were added to each aliquot respectively. The mixtures were incubated at 4°C, then equal amounts of anti-CK18 antibody were added. The precipitates were subjected to Western blots to check PKCε and CK18. Results (Fig. 5c,d) showed that, in control lanes where RNA concentrations were zero, the Western blots detected both PKCε and CK18, indicating that these two molecules bound one another and formed a conjugate. It was interesting that, in the lanes where C/EBPβ 3′UTR RNA was added in increasing concentrations, the amounts of PKCε and CK18 both gradually increased, in line with the increase of added C/EBPβ 3′UTR RNA (Fig. 5c). This indicated that C/EBPβ 3′UTR RNA formed a complex with the PKCε-CK18 conjugate, so that the antibody bound more CK18 (and PKCε) in the presence of more C/EBPβ 3′UTR RNA. To confirm the specificity of the interaction of C/EBPβ 3′UTR RNA with the PKCε-CK18 conjugate, we performed a control RNA binding-coupled immunoprecipitation using β-tubulin RNA instead of C/EBPβ 3′UTR RNA. Result showed that neither positive nor negative correlations existed between the amounts of β-tubulin RNA and the PKCε bound to CK18 (Fig. 5d). Therefore, there is a specific interaction between this 3′UTR RNA, PKCε and CK18.

Based on these facts, we propose that the inhibition of the PKCε activity, and hence the suppression of the SMMC-7721 hepatoma cell growth, was achieved though the formation of a complex by direct physical interaction between C/EBPβ 3′UTR RNA, PKCε and CK18.

## Discussion

PKCε activates many genes, such as RAS [30]–[33] and STAT3 transcription factor, in various signal transduction pathways [34]. PKCε also regulates many cellular proteins by phosphorylation, e.g., it phosphorylates cytoskeleton proteins [35]. Besides this, PKCε plays a role in the carcinogenesis of fibroblast and epithelial cells as an oncogene [36]–[38]. Therefore, PKCε appears to be an important regulating protein kinase, with large areas of function, under both normal and tumoral conditions. Thus, altering the activity of PKCε could lead to various impacts on the essential biological activity of cells. Most relevantly, the inhibition of PKCε activity in malignant cells may result in tumor suppression.

3′UTR has long been recognized as a regulator for the translation of its original mRNA [39]. In recent years, a direct interaction has been found between 3′UTRs and miRNAs acting as regulators [40], [41]. We found that C/EBPβ 3′UTR specifically bound keratin 18, a component of cellular intermediate filaments. In connection with the results presented in this paper, we suggest that the binding of C/EBPβ 3′UTR to CK18 should be considered not only as a means of intracellular localization (for the C/EBPβ mRNA), but also as a means of regulation; the formation of a complex by CK18, PKCε and C/EBPβ 3′UTR, and the decrease in activity of PKCε, may be proof of such a regulatory role. It has been reported that PKCε, both in activated and non-activated form, is capable of binding a large number of cellular molecules [32], [33]. Here we have found, for the first time, that the C/EBPβ 3′UTR also forms a specific complex with CK18-PKCε conjugate. Although it is not clear at the present time, it is reasonable to propose that PKCε and its target factors may also be regulated by the 3′UTR of endogenous C/EBPβ mRNA *in vivo*.

Another factor is that, in our case, we have not found definite evidence that the overexpression of exogenous C/EBPβ 3′UTR in 7721 cells can affect the endogenous expression of C/EBPβ gene. This may evince the complicated mechanism involved in the miRNA regulation of gene expression. Our results indicate that the C/EBPβ 3′UTR, when isolated from its original mRNA, was not inactivated, but, on the contrary, became a factor that executed different functions from its original. Therefore, this may suggest that the function of an isolated 3′UTR represents a novel regulatory function of (at least a part of) eukaryotic mRNA; and the functional independence of the 3′UTR segment may imply that it is possibly derived from some primordial, ancestral RNA. This awaits further investigation.

To conclude, our results suggest that tumor suppression of SMMC-7721 human liver cancer cells by C/EBPβ 3′UTR is caused by inhibiting PKCε activity through the specific binding of C/EBPβ 3′UTR RNA to PKCε and CK18. This may be a novel regulatory function of at least a portion of eukaryotic mRNAs. Beside these findings, our work has raised many additional problems. For example: which mRNA 3′UTRs have tumor suppression functions? Does the same molecular mechanism underlie the function of other known tumor suppressor 3′UTRs? Does the 3′UTR of any intact mRNA form part of the structure of a molecular machine? Further investigation of these problems is required to gain a better understanding of the detailed biological regulatory functions of these mRNA elements, as well as to explore new therapeutic strategies to more effectively combat cancer.

## Materials and Methods

### Cell culture

The SMMC-7721 cells were from the Cell Bank of Chinese Academy of Sciences, Shanghai, and were kept in a CO_2_ incubator in RPMI1640 medium containing 10% newborn calf serum (top grade, Si Ji Qing Biotechnological Materials Co., Hangzhou) supplemented with 100 µg/ml of ampicillin and 100 units/ml of streptomycin sulfate. The culture conditions for the Cl1 cells (21) were the same, except that 400 µg/ml of G418 (Invitrogen) was periodically added to the medium to maintain the purity of the Cl1 cells.

### Western blotting

Before mixing with the loading buffer, all operations were done at 0–4°C. The cells treated according to experimental requirements were collected with trypsinization, washed with cold PBS, then suspended in 1X PAGE loading buffer, and dissolved by heating for five minutes at 90–100°C. When necessary, cells were dissolved directly in dishes or plate wells with RIPA, then mixed with the PAGE loading buffer. The cellular lysates were separated on 10% SDS-PAGE with prestained molecular weight markers (Fermentas). The protein bands in the gels were transferred to a PVDF membrane (Millipore) using Trans-Blot Apparatus (Bio-Rad) according to the manufacturer's instructions. The membranes were blocked in Tris-5% nonfat milk powder for 1 h, then incubated in the milk solution with the primary antibody at room temperature for 2.5 h or at 4°C overnight. This was followed by 2 washes of 10 minutes each in 5% nonfat milk, and then the membrane was incubated with the horseradish peroxidase (HP)-conjugated secondary antibody in 5% nonfat milk for 1 h at room temperature. After 4 washes in Tris-buffered saline-Tween (TBS-T), the blots were subjected to the Luminol chemiluminescence kit (Santa Cruz) and detected with a LAS4000 System (FujiFilm).

### Transient RNA transfection

RNA was transiently transfected into 7721 cells grown in multiwell plates or 35 mm diameter dishes, using Lipofectamine 2000 (Invitrogen), according to the manufacturer's instructions. Transfections were done when the cells had grown to about 30–50% confluence. The amounts of transfected RNA were determined by the requirements of each individual experiment; generally, about 0.5 µg RNA was used for each well of a 96-well plate. For larger culture devices such as 24-well plates or 35-mm diameter dishes, the amount of RNA could be as much as 5 µg for each well or dish. The cells were subjected to Western blotting at scheduled times (ranging from 1/2 h to about 42 h posttransfection); immunofluorescent and molecular beacon staining were performed 24–48 h posttransfection.

### Immunocytochemical fluorescent and molecular beacon staining

The cells (including RNA-transiently transfected cells) were grown on microscopic coverslips placed in 12-well plates until they reached about 80% confluence (24–48 h). Then the cells were washed twice with PBS and fixed with 4% paraformaldehyde at room temperature for 30 min, followed by a further wash with PBS. The cells were treated with 0.5% Triton X-100 for 5 min,washed thoroughly with PBS, and treated with 5% nonfat milk for 1 h. The blocked cells were washed three times with PBS, with each wash lasting 5 min, and the monoclonal antibody working against CK18 (Zymed and Santa Cruz, 1∶200) was added. The cells were incubated at 4°C overnight. On the following day, the cells were washed with PBS three times, with each wash lasting 10 min, then the FITC-labeled secondary antibody(Zymed and Santa Cruz, 1∶150–200)was added and the cells were incubated at room temperature for 1 h. The stained cells were washed three times with PBS, with each wash lasting 5 min, stained with DAPI (Sigma) for 5 min, and washed three times with PBS, with each wash lasting 5 min. Finally the stained cells were sealed on their coverslips and observed.

The molecular beacon specific to C/EBPβ 3′UTR RNA (sequence: 5′-CAGCGAGCCGGGC- CCTGAGTAATCGCGCTG-3′. Modification: 5′:-HEX; 3′: -DABCYL) was designed and synthesized in Shanghai ShineGene Molecular Biotechnology Co., China. The concentration of the molecular beacon used for staining was 1–10 µg/ml; the concentrations for experimental and control groups were strictly identical. The molecular beacon was added to cells which had been fixed with a 4% paraformaldehyde solution, and the cells were incubated at 75°C for 10 min followed by incubation at room temperature for 1 h, then washed with PBS three times, with each wash lasting 10 min, and treated with 5% nonfat milk for 1 h. The immunofluorescent staining was done subsequently as above. The molecular beacon-stained and immunostained cells were observed in a Leica TCS SP2 Confocal Microscope System (Leica Microsystems).

### Protein kinase inhibition

Individual components from the SCREEN-WELL small protein kinase inhibitors library (Enzo(Biomol)) were added, in various concentrations according to relevant literature, to the culture media of 7721 cells grown in 96-well plates. To improve cell permeability, 50 µg/ml saponin (Merck; kindly provided by Rong-Gui Hu) was added into the cell culture together with the inhibitors. After scheduled times (1/2 h –42 h) the cells were lysed, separated on PAGE and subjected to Western blotting to detect CK18 and pCK18. Control Cl1 cells were cultured in the same plate in the same medium without inhibitors.

### *In vitro* protein *kinase Cε activity assays*

In the preliminary test of the activity of calcium- independent PKCs, M-phase-enriched SMMC-7721 cells were homogenized at 0–4°C in a lysis buffer (50 mM Tris-HCl pH 7.5/10 mM MgCl_2_/2 mM EGTA/0.01% Brij 35/0.2% dodecyl dimethyl betaine) (Chu Xing Chemical Industries, Ltd., Shanghai)/1 X protease inhibitor cocktail (AppliChem)/0.5–1 µ/µl RNasin) and centrifuged at 12000 g for 30 min at 4°C. 10 µl of the supernatant was mixed at 0°C with 1 µl of ATP mixture (2 µl H_2_O, 1 µl 1 mM ATP/100 mM MgCl_2_, 3 µl 5′[γ-^32^P]ATP (10 µCi/µl, 3000 Ci/mmol, Fu Rui Co., Beijing, China)) and 1 µl H_2_O or C/EBPβ 3′UTR RNA in increasing concentrations. The mixtures were incubated at 30°C for 30 min, then PAGE loading buffer was added and the samples were separated with SDS-PAGE, after which the gels were dried and autoradiographed.

In the *in vitro* protein kinase Cε assay, one 100-mm dish of SMMC-7721 cells at 70–80% confluence was cultured in 1640 medium without serum overnight, after which cell extract was prepared by homogenization and centrifugation as above. Another 100-mm dish of SMMC-7721 cells, cultured in complete 1640 medium containing serum, was used to enrich CK18 using Bronfman's method (28): the E fraction obtained by this method was centrifuged at 12000 g, and the precipitate was used for enriched CK18. The two preparations were mixed at 0°C, okadaic acid (Calbiochem) was added to 0.04 µM, and PMA (Calbiochem; kindly provided by Xiao-Hui Zhang) was added to 0.5 µM. The mixture was divided into Eppendorf tubes at 0°C, with 8 µl in each tube, and 1 µl of 10 mM ATP/10 mM EGTA was added to each tube. Then 1 µl of C/EBPβ 3′UTR RNA, or 1 µl of εV1-2 (kindly provided by Zhi-Qi Zhao), both in increasing concentrations, were added to the respective tubes. The tubes were incubated at 30°C for 20–25 min. The samples were mixed with PAGE loading buffer, separated on 10% PAGE, and subjected to Western blotting for pCK18 and CK18.

### RNA binding-coupled co-immunoprecipitation

SMMC-7721 cells, grown in multiwell plates, were lysed at 0°C in a keratin lysis buffer(1 X PBS pH 7.4,5 mM EDTA, 0.1 mM PMSF, 1% protease inhibitor cocktail, 2% dodecyl dimethyl betaine,0.04 µM okadaic acid) and centrifuged at 12000 g and 4°C for 15 min. 10 X Binding buffer (400 mM KCl, 30 mM MgCl_2_, 100 mM HEPES pH 7.8, 10 mM DTT) was added to the supernatant to form a final concentration of 1 X. The mixture was equally divided into Eppendorf tubes, and C/EBPβ 3′UTR RNA or control β-tubulin RNA (an 1.4 kb coding region segment in the same volume with increasing concentrations) was added to each tube respectively. After incubation at 4°C for 1 h, equal amounts of anti-CK18 antibody were added to each tube, and the incubation at 4°C continued for 2.5 h. The antibody against IgG was then added and incubation lasted overnight. The reaction mixtures were centrifuged at 12000 g for 30 min at 4°C, the precipitates were subjected to PAGE and Western blotting to detect CK18 and PKCε. The bands of the IgG light chain at about 25 kDa from the antibody against CK18 were used as an inner control.
